# The mushroom body development and learning ability of adult honeybees are influenced by cold exposure during their early pupal stage

**DOI:** 10.3389/fphys.2023.1173808

**Published:** 2023-04-20

**Authors:** Chenyu Zhu, Han Li, Xinjian Xu, Shujing Zhou, Bingfeng Zhou, Xiang Li, Hongzhi Xu, Yuanmingyue Tian, Yanxin Wang, Yu Chu, Xianlan Zhang, Xiangjie Zhu

**Affiliations:** ^1^ College of Animal Science (College of Bee Science), Fujian Agriculture and Forestry University, Fuzhou, China; ^2^ Honeybee Research Institute, Fujian Agriculture and Forestry University, Fuzhou, China

**Keywords:** *Apis mellifera*, pupae stage, cold-temperature stress, association learning, development

## Abstract

The honeybees are the most important pollinator in the production of crops and fresh produce. Temperature affects the survival of honeybees, and determines the quality of their development, which is of great significance for beekeeping production. Yet, little was known about how does low temperature stress during development stage cause bee death and any sub-lethal effect on subsequent. Early pupal stage is the most sensitive stage to low temperature in pupal stage. In this study, early pupal broods were exposed to 20°C for 12, 16, 24, and 48 h, followed by incubation at 35°C until emergence. We found that 48 h of low temperature duration cause 70% of individual bees to die. Although the mortality at 12 and 16 h seems not very high, the association learning ability of the surviving individuals was greatly affected. The brain slices of honeybees showed that low temperature treatment could cause the brain development of honeybees to almost stop. Gene expression profiles between low temperature treatment groups (T24, T48) and the control revealed that 1,267 and 1,174 genes were differentially expressed respectively. Functional enrichment analysis of differentially expressed genes showed that the differential expression of *Map3k9*, *Dhrs4*, and *Sod-2* genes on MAPK and peroxisome signaling pathway caused oxidative damage to the honeybee head. On the FoxO signal pathway, *InsR* and *FoxO* were upregulated, and *JNK*, *Akt*, and *Bsk* were downregulated; and on the insect hormone synthesis signal pathway, *Phm* and *Spo* genes were downregulated. Therefore, we speculate that low temperature stress affects hormone regulation. It was detected that the pathways related to the nervous system were Cholinergic synapse, Dopaminergic synapse, GABAergic synapse, Glutamatergic synapse, Serotonergic synapse, Neurotrophin signaling pathway, and Synaptic vesicle cycle. This implies that the synaptic development of honeybees is quite possibly greatly affected by low temperature stress. Understanding how low temperature stress affects the physiology of bee brain development and how it affects bee behavior provide a theoretical foundation for a deeper comprehension of the temperature adaptation mechanism that underlies the “stenothermic” development of social insects, and help to improve honeybee management strategies to ensure the healthy of colony.

## 1 Introduction

As the most important pollinating insect, honeybees have brought great economic benefits to human beings in terms of pollinating crops and maintaining ecosystem stability (Bareke and Addi., 2019; Reilly et al., 2020). However, many factors threaten the survival of honeybees, such as poor nutrition (Martelli et al., 2022), parasites (Wu et al., 2022), pesticides (Wang et al., 2022), and nest temperature. Management strategies of nest temperature is one of the factors causing the decline of colony populations (Nazzi and Le Conte, 2016). The ability to adapt to a particular temperature range is essential for the survival of all living beings. The temperature of the environment has a significant impact on the growth and development of honeybees, their colony activities, individual behavior, and ability to resist disease (Irwin and Lee, 2000; Stabentheiner et al., 2021). Especially, when experiencing cold stress in early spring, colonies usually struggle to regulate nest temperature for their stenothermic broods. Particularly the small population sized colonies, tend to shrink during cold waves, leading to the broods distributing the outer edge of the hive exposed to cold without protection (Simpson, 1961; Becher et al., 2009; Stalidzans et al., 2017). These cold damaged broods will be groomed out of the colony consequently. The development of honeybee broods determines the health level of colonies, which affects the quality and yield of colony products (Zhou, 2002).

Temperature is a crucial ecological factor that significantly affects the individual development of honeybees. Studies have shown that thermal stress can have negative effects on bee brood development, both in laboratory and field settings (Zhao et al., 2021). Compared to most insects, the developmental temperature range of honeybees is much narrower, so that they are more susceptible to temperature fluctuations. The temperature range of broods areas varies within a narrow temperature range of 32°C–36°C (Kleinhenz et al., 2003). Research has shown that the temperature ecological amplitude for honeybee eggs and pupae’s development is between 29°C and 38°C, as reported by several studies (Medrzycki et al., 2010; Zhu et al., 2006; Es'kov, 1998), which is indeed a narrower temperature range. Furthermore, to ensure development of honeybee broods, temperature requirements are more stringent (Kronenberg and Heller, 1982; Jones et al., 2004). The optimal development temperature is 35°C to complete their metamorphosis from eggs to larvae, pupae, and finally to eclosion (Seeley, 1983; Seeley and Visscher, 1985). A study reported that the capped broods cultured at constant temperature of 33°C exhibited poorer dance behavior performance and lower homing rates after emergence, while those cultured at 30°C were unable to learn the set feeder collection (Tautz et al., 2003). Another study showed that incubating capped broods at constant temperature at 29°C–33°C led to a significant decrease in the number of synaptic complexes in the lip region of the brain mushroom of honeybee, which may explain their poor learning and memory abilities (Groh et al., 2004). Additionally, exposure to low-temperature during the capped brood stage can lead to increased pupal mortality, misorientation, adult mortality, and the abnormal wing veins in honeybees (Wang et al., 2016; Zhu et al., 2018).

The pre-pupal stage of honeybees is highly susceptible to low temperatures, as noted by Wang et al. (2016). A study on the differential expression of genes under low temperature stress at 20°C revealed that western honeybees pre-pupae and pupae primarily regulate their development process through hormones to adapt to low temperatures by slowing down or halting their development (Yao et al., 2020; Li et al., 2022; Liu et al., 2022). Of all the pupal stages, early pupa stage is most vulnerable to low temperature treatment for 12–48 h, resulting in the highest mortality rate and a reduction in the longevity of emerging bees by 30%–60% (Wang et al., 2016). This makes it an ideal sample for studying the effects of low temperature on honeybee development and behavior. This paper investigates the effects of low temperature on honeybee pre-pupae mortality, brain tissue morphology, learning ability of adult individuals, and the transcriptome. The results will provide not only a theoretical understanding of the stress on brain development and brain function in honeybees, but also can be used to assess the negative effects of early stress on later life quality. Specifically, brood experienced with cold stress are likely to exhibit elevated mortality rates, sub lethal brain dysfunctions leading to colony decline and failure.

## 2 Material and methods

### 2.1 Sample preparation

In spring, the western honeybee (*Apis mellifera*) colonies were sampled. To obtain consistent honeybee eggs, the queens were confined to laying eggs on a single frame individually. These eggs were permitted to mature in the colony until they were capped. The patches of comb containing newly capped broods within 4 h were excised and placed in a chamber with a constant temperature and humidity (35°C ± 0.2°C, RH 75%) [THB-250, Allison Instruments and Equipment (Shanghai) Co., Ltd., precision ± 0.1°C] for 4 days (Wang et al., 2016).

### 2.2 Mortality and investigating odor-associated behavior

The study involved treating 4-day-capped brood cells at 20°C (RH 60%, CTHI-250B, Stik Group LLC, United States; precision, ±0.1°C) for varying durations of 12, 16, 24, or 48 h, denoted as T12, T16, T24, and T48 respectively. Except during cold stress, brood cells were incubated at 35°C. The control group comprised 110 samples, while T12, T16, T24, and T48 had 49, 65, 59, and 51 samples respectively. The difference in mortality rates between the treatment and control groups was determined using the chi-square test (SPSS 16.0). Newly emerged bees were transferred into the cages, and incubated at 33°C and 50% RH for 7 days before initiating behavioral studies. The honeybees were fed pollen candy made from canola pollen mixed with syrup (50%) and provided with pollen/syrup (50%)/water *ad lib*, which was replaced every 2 days. The study on associative learning involved 211 participants grouped into control, T12, T16, and T24, with sample sizes of 108, 35, 48, and 20 individuals respectively. The experiment was repeated three times, and due to an elevated mortality rate in the T24 group, the available sample size for measuring learning behavior was limited.

The experiment involved the use of individual honeybees that were secured in copper tubes with cloth tape, allowing their proboscis to move freely. The bees’ antennae were exposed to a solution containing 50 mg/mL of syrup, and those that did not respond by extending their proboscis were excluded from the experiment. The bees were then starved for 2 h at a temperature of 30°C with a relative humidity of 60%, after which an association learning test was conducted.

During the test, the honeybees were exposed to the odor of nonyl alcohol for a period of 3 s, followed by a 4-s stimulation of 50% sugar water. The interval between each training process was 10 min, and each worker bee repeats the training three times. When only stimulated by the odor, if the worker bees could extend their proboscis, it was recorded that the smell association learning was successful (Müller, 2002; Felsenberg et al., 2011).

### 2.3 Preparation and observation of tissue section

The treatment groups for further sectioning observation were comprised of the samples that exhibited poor performance in the odor association behavior experiment, namely, T24 and T48. Meanwhile, three control groups were established, consisting of brood cells from 4-day-old, 5-day-old, and 6-day-old capped broods that were incubated at 35°C These control groups were labeled as Control, Control 24 and Control 48, respectively. In order to prepare the specimens for observation, the bees’ heads were severed and subjected to paraffin sectioning. Subsequently, the hematoxylin-eosin staining method was employed, as per the protocol described by Estrada et al. (2005).

### 2.4 Transcriptomics methods

#### 2.4.1 The sample preparation

The transcriptome analysis sample preparation method remains consistent with the procedure outlined in Section 2.1. The experimental conditions involve subjecting the samples to low temperature treatment for 24 and 48 h, referred to as T24 and T48, respectively. The HE section documents the morphological changes observed in various parts of the honeybee brain during the early pupal stage. The brain tissue morphology of the samples treated with low temperature was found to be more similar to the pre-treatment state, indicating that low temperature resulted in the cessation of tissue differentiation. Consequently, the control group was selected from samples with a similar developmental status to those subjected to low-temperature treatment. To ensure the representativeness of the samples, each group comprised 100 bees and three biological replicates.

#### 2.4.2 High throughput sequencing and sequencing data quality control

The Illumina HiSeqTM 4000 platform was employed by Guangzhou Chidio Biotechnology Co., Ltd., to construct each sample library and carry out high-throughput sequencing. The obtained offline data was filtered with fastp, resulting in clean reads after removing reads containing adapters, reads with over 10% unrecognized bases, reads with more over 50% adenine content, and reads with a mass value Q ≤ 20. The raw data was compared to the genome of *A. mellifera*, Amel_ HAv3.1 (NCBI Assembly: GCF_003254395.2), and deposited in the NCBI SRA database with accession numbers SRR15710549–SRR15710554.

#### 2.4.3 Screening and assessing the functionality of DEGs

Utilizing DESeq2 software, the difference between the treatment group and the control group of biological duplicates (Love et al., 2014) was investigated. The DEGs (Differentially Expressed Genes) were identified with a screening condition of |log2FC| ≥ 1 (FC, Fold Change = FPKM of the treatment group/FPKM of the control group) and FDR ≤ 0.05. The DEGs of T24 vs. Control, T48 vs. Control were analyzed individually to obtain the DEGs after low-temperature treatment. Following this, the further GO functional classification and KEGG pathway analysis of DEGs were conducted.

#### 2.4.4 Real-time fluorescence quantitative PCR

The primers used in this study were designed using NCBI Primer Blast (listed in Table 1). Each sample was subjected to three biological replications and three technical replications of the target gene. Total RNA was extracted from the samples using Transzol Up Plus RNA Kit, and cDNA was synthesized through reverse transcription. The resulting cDNA was utilized as a template for quantitative PCR (qPCR), and the reaction system (10 μL) was performed according to the instructions provided with the *PerfectStart*™ Green qPCR SuperMix. The relative expression of the target gene was determined using the 2^−△△Ct^ method (Livak and Schmittgen, 2001), with Actin serving as the internal reference gene. The mean value of each gene’s relative expression was transformed using log_2_. The difference in gene expression between the treatment group and the control group was analyzed using an unpaired *t*-test to determine statistical significance.

**TABLE 1:** Primers for qPCR of DEGs.

Gene	Gene ID	Gene description	Primer sequence
*Actin*	*LOC413144*	Actin related protein 11	F:TGCCAACACTGTCCTTTCTG
			R:AGAATTGACCCACCAATCCA
*Mblk-1*	*LOC 408521*	Mushroom body large-type Kenyon cell-specific protein 1 isoform X1	F:AACACCAAATACGACCCAAAAC
			R: CAA​CAG​AGC​CTT​CTC​CAC​TTC​T
*l(2)efl*	*LOC 412197*	Protein lethal(2)essential for life-like	F: GCG​TCG​ATA​TGT​GCT​TCC​AC
			R: GCT​GCC​TTA​ATA​GCT​GGT​GC
*Orct*	*LOC 412056*	Organic cation transporter protein-like isoform X1	F: TAG​TTG​ATC​GCT​GTA​GCT​CCC
			R: CCT​TGG​GAA​ACG​AAC​GTG​AAT
*FoxP*	*LOC 408423*	FoxP protein isoform X17	F: GCG​CAC​ATT​ACA​CGG​CAT​TT
			R: GGG​TTG​TTG​GAG​AGA​CGG​TT
*FoxO*	*LOC 727091*	Forkhead box protein O isoform X2	F: CGC​TCG​CTT​CTC​CAT​TAT​CCT
			R: GGC​AGT​AGG​AAC​TGA​CCG​AG

## 3 Results

### 3.1 Mortality

**FIGURE 1:**
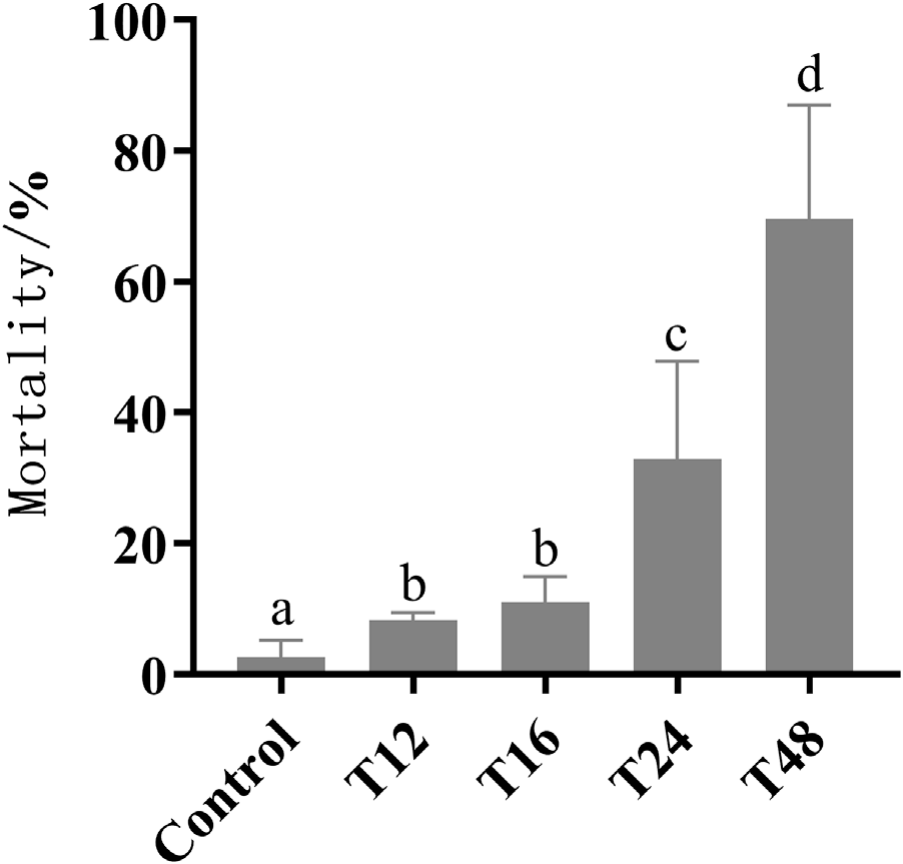
The sample size of control group, T12, T16, T24, and T48 were 110, 49, 65, 59, and 51 respectively. Mortality (mean value ± standard deviation, ‾X ± SD) of early pupa experienced with 20°C for 12, 16, 24, and 48 h. The different letters in lower case indicated significant difference *p* < 0.05 level.

Prolonged exposure to low temperature during capped brood resulted in increased mortality. Mortality rates were found to be significantly higher in groups exposed to cold duration of 12, 16, 24, and 48 h, with rates of 8.25%, 11.06%, 32.9%, and 69.6%, respectively, compared to the control group (2.61%). The highest mortality rate of 70% was observed in the 48-h exposure group across all low-temperature treatments (Figure 1). Unfortunately, a few newly-emerged honeybees died prematurely, preventing them from undergoing an association learning ability test after 7 days.

### 3.2 Association learning ability

**FIGURE 2:**
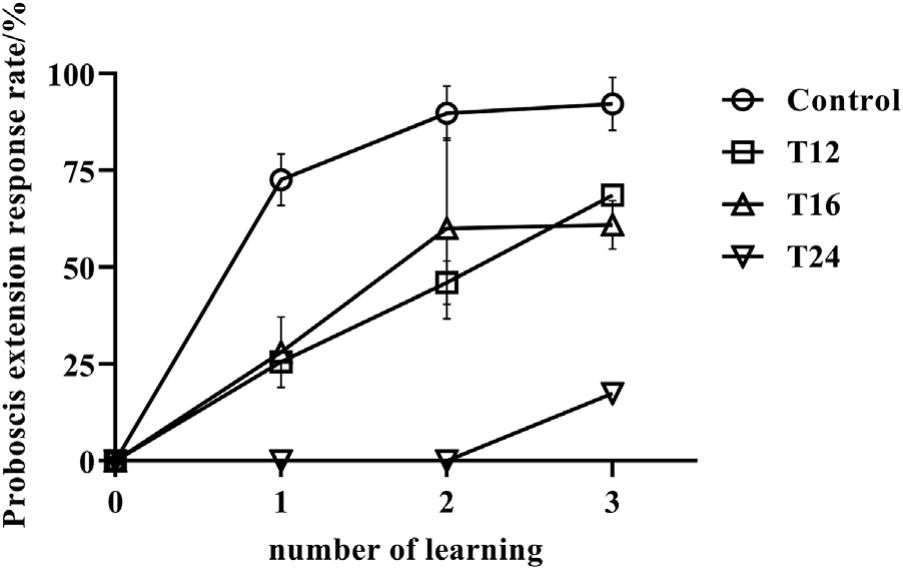
The association learning success rate (mean value ± standard deviation, ‾X ± SD) of adult honeybee PER after low-temperature treatment. Note: The sample size of control group, T12, T16, and T24 were 108, 35, 48, and 20 respectively. It has been observed that low temperature treatment at the early stage of pupae can have an effect on the learning behavior in the emerged adult bees. Prolonging the treatment time leads to a decrease in their learning ability. A significant difference was found between the control group and the low temperature treatment groups (*p* < 0.05). The control group showed stronger learning abilities and faster learning, while the low temperature treatment group required three times of training, and only a few individuals were able to learn. Moreover, the prolonged treatment time of 48 h led to an increased number of bees unable to emerge, and those that did had a short lifespan, most of them dying within 7 days and thus unable to have their learning abilities tested.

The results indicate that the mean rate of proboscis extension response in adult bees exposed to cold treatment was significantly lower than that of the control group, as depicted in Figure 2. Furthermore, an increase in processing time led to a decrease in their learning ability, as evidenced by the proportion of final learning and the number of learning trials required to achieve that level of learning.

At the beginning of the experiment, when the four groups of samples were exposed to odor, none of the bees in any group exhibited a proboscis extension response, indicating that none of the samples were preferred in the odor association behavior experiment. The results of the study showed that the control group of honeybees had a learning rate of 72.61% after the initial learning phase, which increased to 89.80% and 92.23% in the second and third learning phases, respectively. This suggests that the majority of the control group honeybees successfully learned the task. In contrast, the honeybees exposed to low temperatures (T12 and T16) showed a low learning rate of 25.7% and 28.2%, respectively, after the first learning phase. Their learning rate improved in subsequent phases, reaching 46.1% and 60.0% after the second learning phase, and 68.6% and 61.0% after the final learning phase. However, their learning rate remained lower than that of the control group (*p* < 0.01). Specifically, honeybees exposed to low temperatures for 24 h had a reduced learning capacity, and after two learning phases, all individuals were unable to form an associative memory between the odd odor and sugar water. The success rate after three learning phases was only 15%, significantly lower than that of the control group and the treatment groups subjected to 12–16 h of low temperatures (*p* < 0.01). These findings demonstrate the negative impact of low temperatures on the learning abilities of honeybees.

### 3.3 Brain section

**FIGURE 3:**
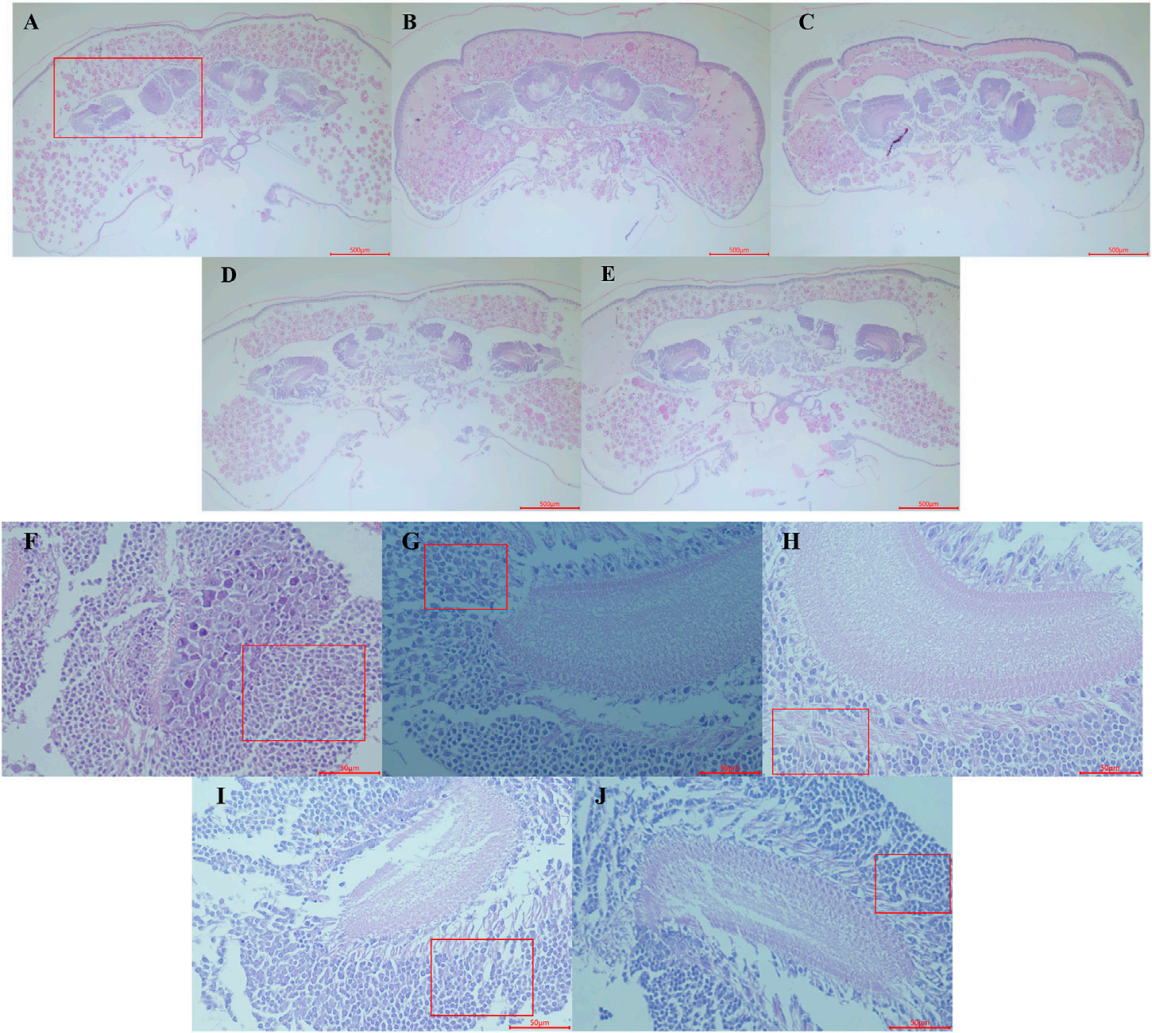
Observation of Haematoxylin and Eosin staining paraffin section of the early pupa of honeybee head. **(A–C)** represents the head section of the control group on the first, second and third days of pupal stage (control groups, 20×). **(D,E)** represents the head section of the first day pupal in the treatment group for 24 and 48 h respectively. **(F–H)** represents mushroom body (red box area in **(A)**) sections of the first, second and third days pupal (control groups, 400×); **(I,J)** represents the mushroom body area section of the first day pupal in the treatment group for 24 and 48 h respectively.

Hypothermia has been observed to hinder the proper development of the brain by causing slow or stagnant brain growth. In healthy bee pupae, brain development usually progresses with age, resulting in more defined regions and clearer boundaries for each part of the brain, alongside an increase in the number of cells (Figures 3A–C). Conversely, honeybee pupae subjected to low-temperature treatment displayed a level of tissue differentiation and cell count similar to that of untreated samples (Figures 3D, E).

Furthermore, low-temperature treatment also affected the development of the honeybee’s mushroom body, which typically grows largeer and becomes more distinct with age, while nerve cell count decreased, and differentiation became more apparent (Figures 3F–H). In contrast, the mushroom body of the samples treated with low temperature for 24 and 48 h exhibited slower differentiation speed, with relatively dense neuroblasts still presented (Figures 3I, J).

### 3.4 Effect of low temperature on transcriptome

The average number of raw read segments for Control, T24 and T48 after sequencing was 42668763, 46607571, and 42077939 respectively. After filtering, the average number of high-quality effective clean reads was 42541931, 46451527, and 41938079 respectively, with the proportion of high-quality effective read segments being greater than 99%. This suggests that the sequencing quality was satisfactory and could be used for further analysis. Additionally, the average number of Q20 and Q30 at both ends was 97.42% and 93.20% respectively (Supplementary Table S1).

#### 3.4.1 Gene ontology (GO) functional analysis

**FIGURE 4:**
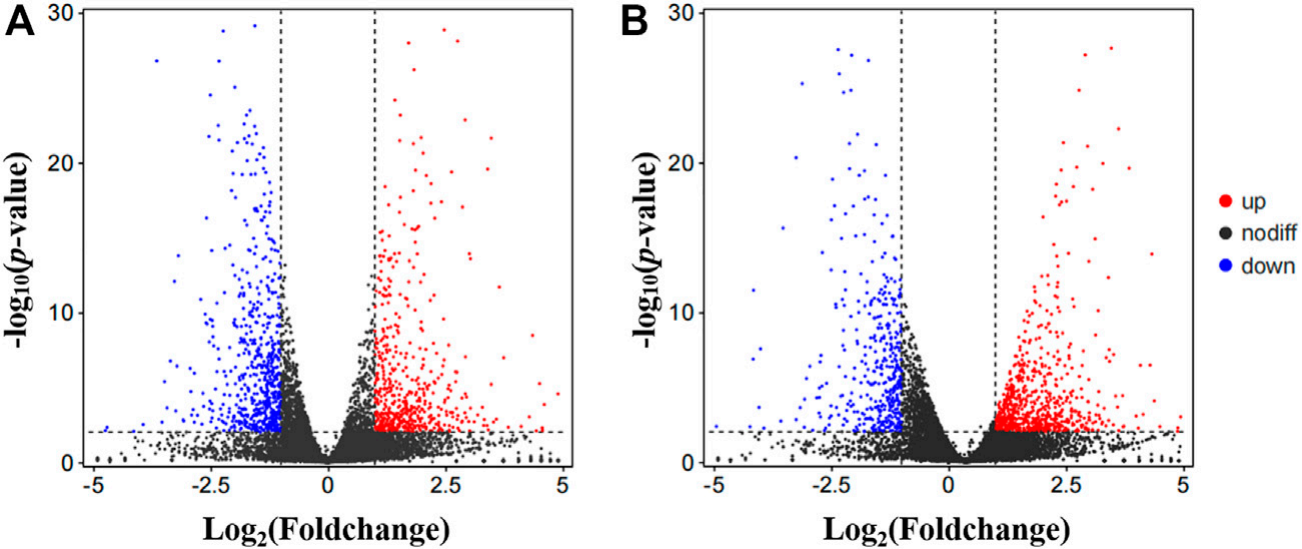
Visualized by volcano plot of differentially expression genes (DEGs). **(A)** Control vs. T24. **(B)** Control vs. T48. Nodiff: genes without significant expression difference; Up, Upregulated DEGs; Down, Downregulated DEGs.

At the early pupa stage, a total of 1,267 and 1,174 genes were differentially expressed in the head after 24 and 48 h of low temperature stress at 20°C respectively (Figure 4).

**FIGURE 5:**
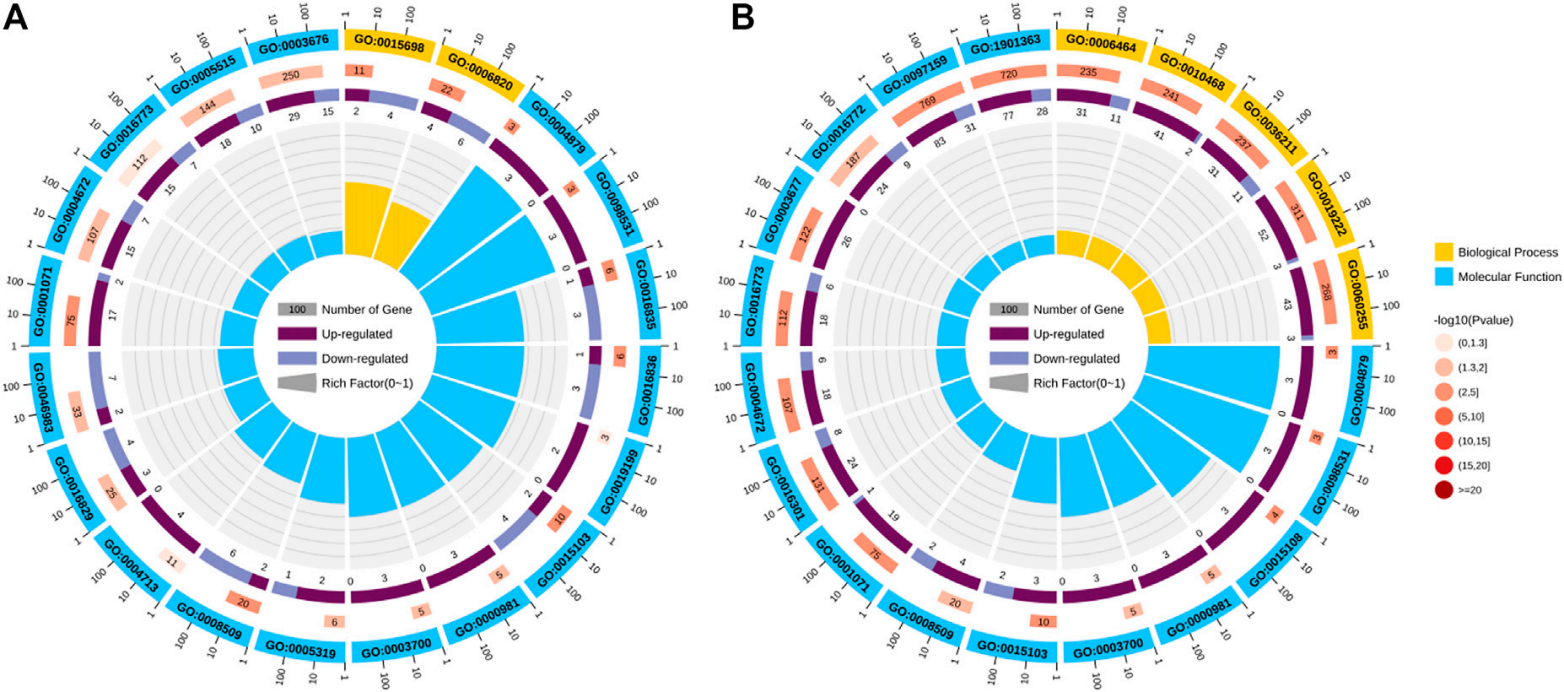
Significant GO terms enriched by differentially expression genes (DEGs). **(A)** Control vs. T24. **(B)** Control vs. T48. The Significant GO term analysis of the DEGs falls into the following GO categories: biological process and molecular function.

24 h: These differentially expressed genes are significantly enriched in seven GO molecular functions, which were inorganic anion transmembrane transporter activity, RNA polymerase II transcription factor activity, ligand-activated sequence-specific DNA bindingRNA, transcription factor activity, direct ligand regulated sequence-specific DNA binding, anion transmembrane transporter activity, carbon-oxygen lyase activity, hydro-lyase activity, and nucleic acid binding transcription factor activity (Figure 5A).

48 h: Of the 20 GO pathways enriched in these differential genes, 11 were enriched in molecular function, which were inorganic kinase activity, nucleic acid binding transcription factor activity, protein kinase activity, RNA polymerase II transcription factor activity, ligand-activated sequence-specific DNA binding, transcription factor activity, direct ligand regulated sequence-specific DNA binding, DNA binding, organic cyclic compound binding, phosphotransferase activity, alcohol group as acceptor, inorganic anion transmembrane transporter activity, chloride transmembrane transporter activity and heterocyclic compound binding. A total of nine biological processes can be identified, including regulation of metabolic process, regulation of gene expression, cellular protein modification process, protein modification process, regulation of macromolecule metabolic process, inorganic anion transport, macromolecule modification, anion transport and dephosphorylation (Figure 5B).

#### 3.4.2 KEGG path enrichment analysis

**FIGURE 6:**
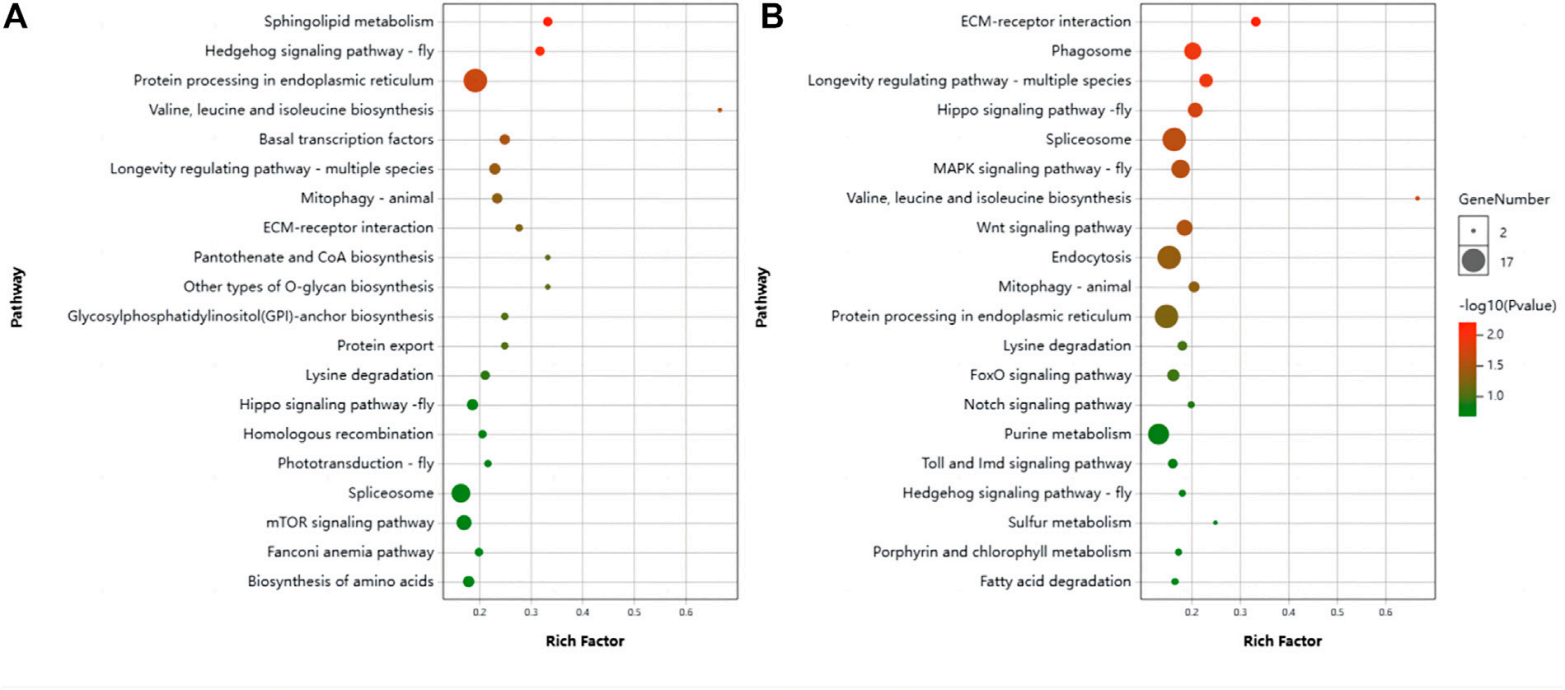
Scatter plot of KEGG pathway enrichment by differentially expression genes (DEGs). **(A)** Control vs. T24. **(B)** Control vs. T48.

24 h: Analysis of the KEGG pathway revealed that the differential genes were significantly enriched in pathways: sphingo-lipid metabolism, Hedgehog signaling pathway—flym, Protein processing in endoplasmic reticulum, Longevity regulating pathway—worm, Valine, leucine and isoleucine biosynthesis, Hedgehog signaling pathway and Basal transcription factorsm (Figure 6A).

48 h: The Pathways significantly enriched were Spliceosome, Endocytosis, MAPK signaling pathway—fly, Focal adhesion, Hippo signaling pathway, Phagosome, PI3K-Akt signaling pathway, Tight junction, Wnt signaling pathway, Hippo signaling pathway—fly, Ras signaling pathway, Insulin resistance, Longevity regulating pathway—multiple species, Insulin signaling pathway, Platelet activation, Oxytocin signaling pathway, Longevity regulating pathway—mammal, Neurotrophin signaling pathway, Adherens junction, Thyroid hormone signaling pathway, Mitophagy—animal, Dopaminergic synapse, Hedgehog signaling pathway, ECM-receptor interaction, Adipocytokine signaling pathway, Valine, leucine and isoleucine biosynthesis (Figure 6B).

The enrichment of pathways related to the nervous system was the same in both treatment groups. They were Cholinergic synapse, Dopaminergic synapse, GABAergic synapse, Retrograde endocannabinoid signaling, Long-term potentiation, Synaptic vesicle cycle, Glutamatergic synapse, Serotonergic synapse, Neurotrophin signaling pathway. This implies that the synaptic development of honeybees is quite possibly greatly affected by low temperature stress.

#### 3.4.3 RT-qPCR

**FIGURE 7:**
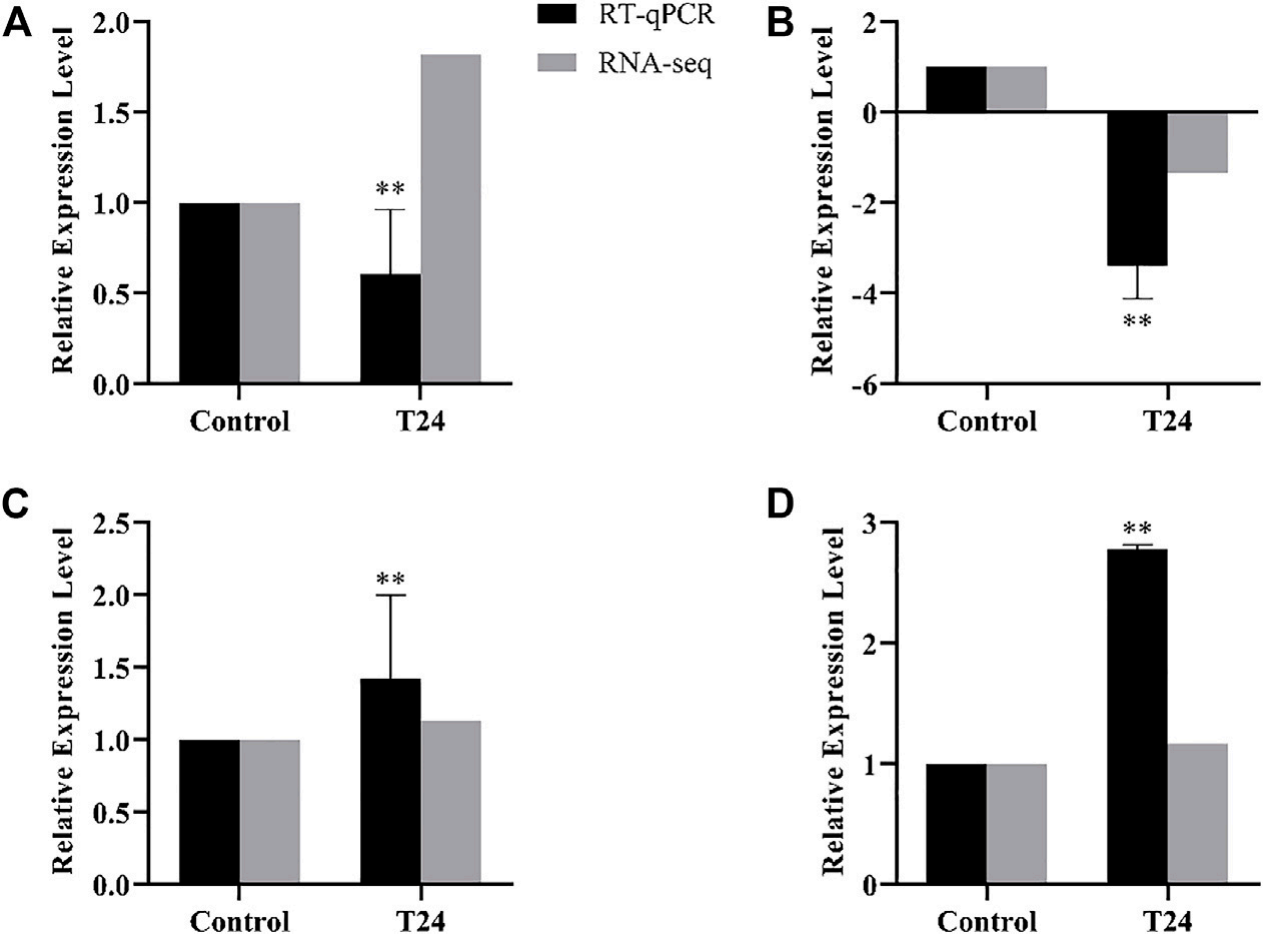
Control vs. T24. RT-qPCR results of RNA-Seq sequencing data of differentially expression genes (DEGs). Data in the figure are mean ± SD, and the asterisk above bars represents extremely significant difference between the treatment group and the control group of the gene relative expression level (*p < 0.05; **p < 0.01; unpaired *t*-test). **(A)**: *Mblk-1*, **(B)**: *l(2)efl*, **(C)**: *FoxP*, **(D)**: *FoxO.*

**FIGURE 8:**
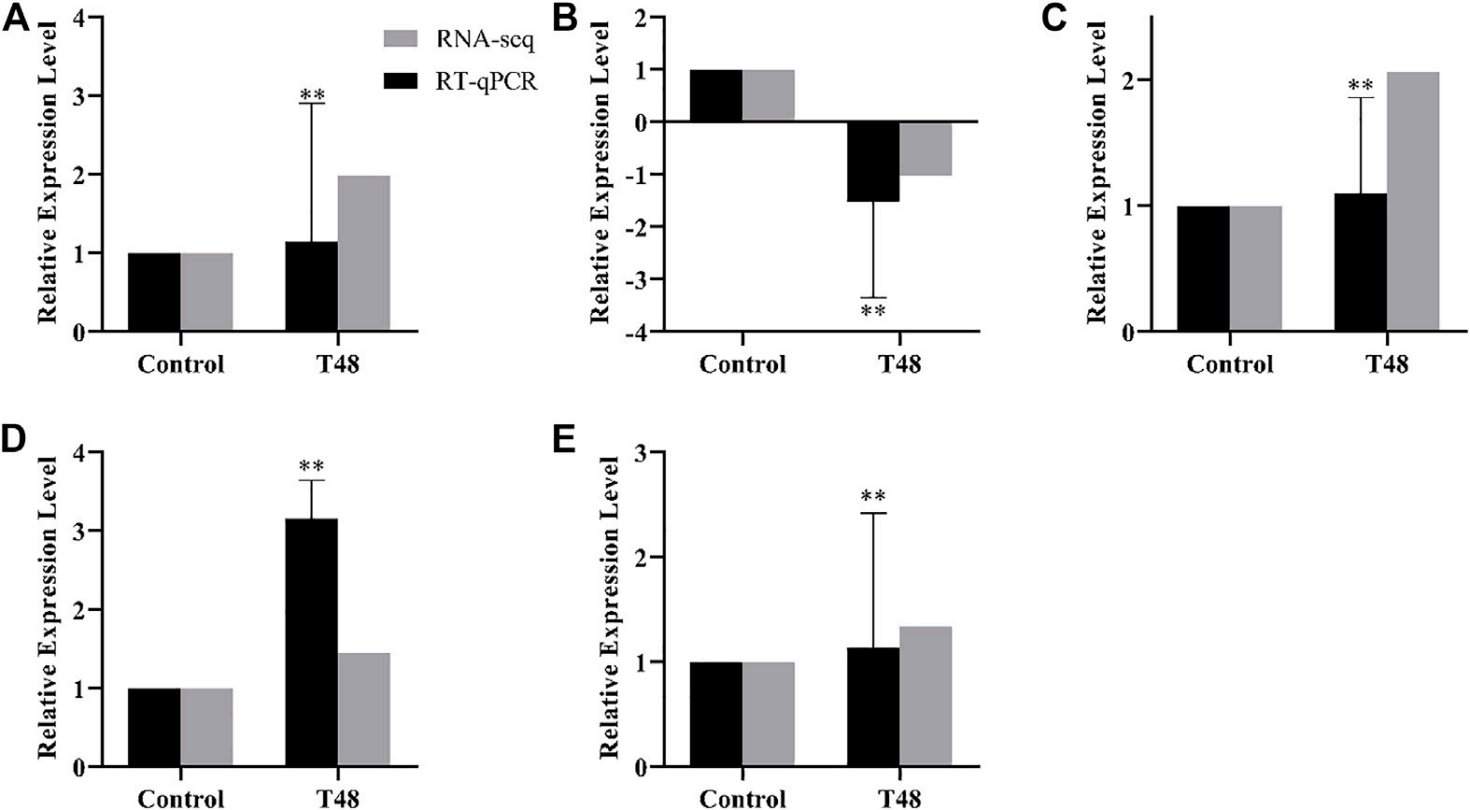
Control vs. T48. RT-qPCR results of RNA-Seq sequencing data of differentially expression genes (DEGs). Data in the figure are mean ± SD, and the asterisk above bars represents extremely significant difference between the treatment group and the control group of the gene relative expression level (*p < 0.05; **p < 0.01; unpaired *t*-test). **(A)**: *Mblk-1*, **(B)**: *l(2)efl*, **(C)**: *FoxP*, **(D)**: *FoxO*, **(E)**: *Orct.*

The difference genes between Control and T24 included *Mblk-1*, *l (2) efl*, *FoxP* and *FoxO* (Figure 7); whereas the difference genes between Control and T48 comprised *Mblk-1*, *l (2) efl*, *FoxP FoxO* and *Orct* (Figure 8). The results indicated that the expression level of these DEGs was consistent with the alteration trend of gene expression level of RNA-Seq sequencing data.

## 4 Discussion

It is widely recognized that honey bee broods are adapted to a stable temperature of approximately 35°C, which is maintained by the adult bees in the colony. For our research, we selected early pupae as our study organism, as this developmental stage is critical for brain development and volume increase. Furthermore, prolonged exposure to cold temperatures resulted in a significant increase in adult mortality, with a mortality rate of 70% observed after 48 h of cold stress. Although exposure to cold temperatures for 12 or 16 h resulted in lower mortality rates, it had sub-lethal effects on pupal development, particularly on the histological development of the mushroom body and the odor-associated learn ability. This led us to predict that sub-lethal effects on honeybee brain development occur when exposed to cold stress. Through comparative transcriptomic RNAseq analysis, we identified genes related to oxidative damage that were regulated during early pupae post cold stress. Overall, our findings provide further insight into the argument proposed by Tautz et al. (2003), Groh et al. (2004), and Becher et al. (2009) that low temperatures can affect bee brain function.

### 4.1 Low temperature treatment affects hormonal regulation in pupal brain development

Previous research has indicated that the delay in the development period caused by cold treatment is highly correlated with the duration of the treatment (Wang et al., 2016). Moreover, the brain sections examined in this investigation indicate that the growth of the brain remains consistent both before and after exposure to cold temperatures. As a result, during the period of cold exposure, it can be inferred that the pupae undergoes a state of stagnation or sluggishness. By integrating transcriptome data and conducting functional cluster analysis of differentially expressed genes, it is inferred that the cold stress triggers an elevation in the juvenile hormone titer while suppressing the synthesis of ecdysone. Furthermore, the low temperature also prevents the transmission of ecdysone signals, thereby regulating the developmental process of honeybees.

An analysis of comparative transcriptome data revealed that exposure to low temperatures for a duration of 24 h resulted in the upregulation of the genes *InsR* and *FoxO*, while expression of the genes *JNK, Akt and Bsk* were downregulated on the FoxO signal pathway. FoxO binds to the *Jheh* promoter sequence, and its upregulation inhibits *Jheh* expression (Zeng et al., 2017), which is responsible for degrading juvenile hormone (JH) and thus regulates its levels (Zhang et al., 2005; Tsubota et al., 2010). This suggests that low temperature stress affects the degradation of JH, resulting in higher JH titer than normal, which helps to maintain honeybee development. *InsR* has the ability to phosphorylate *FoxO*, preventing it from entering the nucleus and causing it to be degraded in the cytoplasm. During insect development, FOXO protein activates the *Br-c* gene downstream of ecdysone (Süren-Castillo et al., 2012; Jin et al., 2013; Cai et al., 2016), which is an essential link for ecdysone to function (Gonzy et al., 2002). This indicated that low temperature may inhibit the function of ecdysone, leading to slowed or halted development. *JNK* plays a critical role in cell stress response, being primarily activated by cytokines and stress from the environment, and participating in various cellular processes such as cell proliferation, differentiation, decay and stress response. The downregulation of *JNK* and its isomer gene *Bsk* due to low temperature affects signal transmission in cells, thus disrupting nueral cell differentiation in pupal brain.

The expression of the *Phm* gene, which is involved in the synthesis of insect hormones, is decreased in response to low-temperature treatment of pupae within the first 24 and 48 h. This gene is linked to the activity of carbon 25-hydroxylase, which controls the production of 20-hydroxyecdysone (20E) (Warren et al., 2004). Additionally, sealing the lid for 48 h after 4 days of low temperature stress results in a decrease in *Spo* (P450 307a1) expression, which is involved in the synthesis of ecdysone (Chávez et al., 2000). This, in turn, affects the synthesis of 20E, hindering the normal operation of the hormone regulatory network. Moreover, 20E in the pupal stage is related to cell proliferation (Bialecki et al., 2002), suggesting that low temperature affects 20E synthesis by affecting the expression levels of *Spo* and *Phm* genes, and thus impairs the development of head cell proliferation. Additionally, the *Kibra* gene on the Hippo signal pathway is upregulated after 48 h of low-temperature treatment. The KIBRA protein and other proteins in the Hippo signal pathway form a KIBRA-MER-EX protein complex to activate Yki phosphorylation, while the phosphorylation of *Yki* prevents it from entering the nucleus (Xiao et al., 2011). *Yki* is associated with the activation of the downstream target gene of 20-hydroxyecdysone (Genevet and Tapon, 2011). Therefore, when low temperature stress causes an increase in the expression of the *Kibra* gene at the head of the cap, the phosphorylation of *Yki* increases, thus inhibiting the downstream target gene of 20E and affecting the role of 20E in cell proliferation (Bialecki et al., 2002; Parvy et al., 2014).

### 4.2 Oxidative damage in pupal brain development under cold exposure

Low temperature could accumulate oxidative damage due to reduced activity of antioxidant enzymes under cold and led to pupal death. Our data showed that the mitogen-activated protein kinase (*Map3k9*, belonging to MAP kinase kinases, MAKKs) gene was upregulated in the T24 group, which is a serine/threonine protein kinase and a key part of the MAPK pathway.

MAPK plays a critical role in transmitting signals from the cell surface to the nucleus, allowing for the transfer of extracellular information to the inside of the cell and adjusting cell activity in response to external stimuli (Santos et al., 2007). The study of *Apis cerana* revealed that silencing the *Mapk9* gene led to an increase in the concentration of malondialdehyde and a decrease in the activity of SOD and POD, causing oxidative damage to cells (Gerber et al., 1998; Santos et al., 2007). Speculation suggests that *Mapk9* expression was increased to resist oxidative damage when the pupae was exposed to low temperature stress. Additionally, the downregulation of *Dhrs4* and *Sod-2* genes in the peroxisome signaling pathway may lead to oxidative damage in bees. Dependent retinol dehydrogenase/reductase (NRDR), as a carbonyl reductase, participates in the redox reaction of various substances in cells (Zhang, 2014). *Dhrs4* gene encodes NRDR, which is located in the plasma membrane of phagocytes and produces active oxygen to eliminate pathogenic microorganisms (Persson et al., 1995). Low temperature stress may inhibit the synthesis of NRDR, impact antioxidant enzymes, and lead to an accumulation of oxidative damage in cells.

Mitochondrial superoxide dismutase 2 (*Sod-2*) counteracts cellular oxidative toxicity by eliminating intracellular reactive oxygen species (Li et al., 1994; Wu, 2010; Xiao, 2015). Research has revealed that a decrease in temperature can result in a rise of active oxygen in insects. To regulate this, insects rely on antioxidant enzymes. The small breasted turtle beetle (*Microdera punctipennis*), a desert insect, enhanced high levels of the antioxidant enzyme *Sod* gene when exposed to low temperatures (Xiao, 2015); likewise, the SOD enzyme activity of Tibetan locust (*Locusta migratoria tibetensis*) in its body wall and digestive tract was increased in response to its body oxidation level when the temperature was low (Wu, 2010). Conversely, when bees were subjected to 24 h of low temperature stress, the expression of the *Sod-2* gene in their heads was downregulated, indicating that the concentration of reactive oxygen species had exceeded the regulatory range of the bee’s antioxidant system, resulting in oxidative damage.

### 4.3 Cell proliferation and apoptosis

Low temperatures can interfere with the usual cell proliferation and apoptosis of honeybee pupa. This study revealed that *Bcl-2* (anti apoptotic gene) and *Diap2* (death related apoptosis inhibitor) of the apoptosis signal pathway were upregulated. These two inhibitors of caspase, which is involved in the proteolysis of cell apoptosis (Roy et al., 1997; Leulier et al., 2006), were found to inhibit the apoptosis of cells in the head of early pupa stage after low temperature stress. The *Akt1* gene, which is closely linked to cell proliferation, was found to be downregulated in the mTOR signal pathway in this study. When the *Akt* gene is silenced, cell proliferation is inhibited, as demonstrated by You et al. (2019). Consequently, a low temperature treatment applied at the early stage of pupa can inhibit the proliferation of bee head cells.

### 4.4 Neural development of honeybee brain

Previous research has demonstrated that honeybee pupae exposed to a constant temperature of 33°C exhibit a decline in their dance behavior and the ability to return to their nest of adult workers (Tautz et al., 2003). Subsequently, researchers investigated the development of the mushroom body of honeybees under the same conditions and discovered that the number of synaptic complexes in the lip area of the mushroom body decreased, and synaptic changes affected neuronal plasticity and behavior (Groh et al., 2004). This study revealed that low temperatures during the pupal stage resulted in reduced number of neuroprogenitor in the mushroom body, which, in turn, impacted the learning ability of honeybees (Figure 2). Additionally, this study detected that pathways related to the development of the nervous system were associated with the development of various synapses. The development of the brain’s mushroom body is closely linked to hormone regulation, oxidative damage, synaptic development, and etc. Consequently, the transcriptome data was meticulously examined to assess the differential expression of genes involved in the nervous system. The following is a discussion of a few key genes.

Results of this study indicated that the cyclic AMP response element binding protein A (*Creb A*) and *Creb-2* (*Atf4*) genes, which are part of the cholinergic synaptic signaling pathway, were found to be upregulated. When neurons in the brain are stimulated, the brain will activate its self-protection mechanism, whereby *Creb* combines with the phosphorylation sites of neuroprotective factors to reduce neuronal damage (Pugazenthi et al., 1999; Marshall R et al., 2000). In response to low temperature stress, *CrebA* and *Creb-2* genes were increased to activate neuroprotective measures and minimize brain damage.

MRJP 1, which accounts for approximately 46% of the water-soluble proteins in royal jelly, has been identified as a major regulatory factor in development (Kamakura, 2011). Research has revealed that the *Mrjp 1* gene is expressed throughout the bee brain, with a highly concentrated presence in the mushroom body of the brain, particularly in the fiber and pericellular area of the intermediate neuron (Peixoto LG et al., 2009). By inhibiting the expression of *Mrjp1* gene, the relative expression of *Mrjp1* gene in honeybee brain was decreased significantly, leading to a marked decrease in learning capacity (Yu et al., 2021). In this study, it was observed that the *Mrjp 1* gene was reduced in the head of early pupae which were subjected to low temperature. It is hypothesized that the cold temperature might be detrimental to the growth of the bee’s nervous system, thus impacting the learning capacity of the bees.

The protein MBLK-1 is expressed predominantly in the large Kenyon cells (LKCs) of pupae and adult bees (Kubo, 2012). This protein plays an important role in metamorphosis downstream of ecdysone signal in the pupal stage and MBLK-1 and P-MBLK-1 take part in the brain morphogenesis during the pupal stage. In the adult stage, *Mblk-1* (*E93*) is involved in synaptic plasticity associated with learning and memory of adult bees (Kubo, 2012; Kumagai H et al., 2020). Consequently, *Mblk-1* has become the criterion for assessing the proliferation of 1KCs, thus representing the development of mushroom body to a certain degree. This has made *Mblk-1* a focus of research. Unpublished data of *Mblk-1* gene expression and protein abundance shows that MBLK-1 was synthesized in a substantial quantity during the early pupal brain development process, peaking on the second day of the early pupal stage and slightly decreasing on the third day, however, still remaining at a high level, suggesting that 1KCs proliferated in this stage. The expression of *Mblk-1* in the low temperature treatment groups increased, yet it could not reach the normal peak. As the low temperature treatment was increased, the expression of *Mblk-1* also increased, suggesting that the 1KCs in the mushroom body continued to develop under the low temperature condition, albeit at a lower rate than the normal proliferation level. This suggests that low temperature treatment inhibited the proliferation of 1KCs. Therefore, further attention should be given to the development of 1KCs in different functional areas of the mushroom body.

## Data Availability

The datasets presented in this study can be found in online repositories. The names of the repository/repositories and accession number(s) can be found below: NCBI SRA database (accession number: SRR15710549–SRR15710554).
