# Supplementary material for: The mushroom body development and learning ability of adult honeybees are influenced by cold exposure during their early pupal stage
Source: Front Physiol. 2023 Apr 20;14:1173808. doi: 10.3389/fphys.2023.1173808 (PMC10157483; doi:10.3389/fphys.2023.1173808)
Supplement: Supplementary file 1 [file Table1.DOCX]

Supplementary Material

**The mushroom body development and learning ability of adult honeybees are influenced by cold exposure during their early pupal stage**

**Chenyu Zhu^1†^, Han Li^1†^, Xinjian Xu^1,2^, Shujing Zhou^1,2^, Bingfeng Zhou^1,2^, Xiang Li^1^, Hongzhi Xu^1^, Yuanmingyue Tian^1^, Yanxin Wang^1^, Yu Chu^1^, Xianlan Zhang^1^, Xiangjie Zhu^1,2*^**

1 College of Animal Science (College of Bee Science), Fujian Agriculture and Forestry University, Fuzhou, China

2 Honeybee Research Institute, Fujian Agriculture and Forestry University, Fuzhou, China

^†^These two authors contributed equally to this study.

*** Correspondence:**

Xiangjie Zhu, [xiangjie_zhu@126.com](mailto:xiangjie_zhu@126.com)

Table S1 The statistics of the transcriptome data of the head of early pupae worker bees exposed to low temperature

| Sample code | Raw reads | Clean reads | Q20 (%) | Q30 (%) |
| --- | --- | --- | --- | --- |
| Control -1 | 43816038 | 43691714 | 97.48 | 93.28 |
| Control -2 | 41521488 | 41392148 | 97.42 | 93.20 |
| Control -3 | 45446176 | 45290538 | 97.65 | 93.61 |
| T24-1 | 59242490 | 59054752 | 97.51 | 93.32 |
| T24-2 | 44436722 | 44281834 | 97.42 | 93.20 |
| T24-3 | 36143500 | 36017996 | 97.64 | 93.70 |
| T48-1 | 42795954 | 43633244 | 97.56 | 93.34 |
| T48-2 | 38622776 | 38543152 | 97.76 | 93.72 |
| T48-3 | 43782086 | 43637842 | 97.62 | 93.41 |

T:Treatment group, the number indicates the duration of the processing. Q20 indicates the number of bases whose sequencing base quality value reaches above Q20 level and its percentage in RawData. The Q20 base sequencing error rate is 1% the Q30 base sequencing error rate is 0.1%.

**
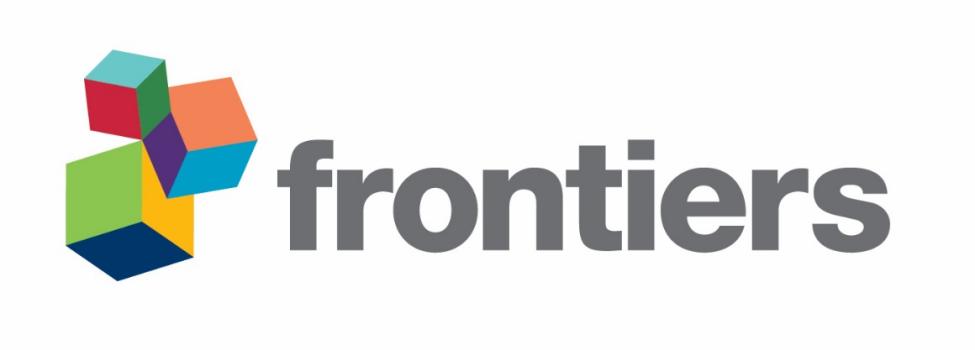
**
